# Dual antiplatelet therapy in acute ischemic stroke: Does diabetes mellitus define a target population?

**DOI:** 10.1016/j.neurot.2026.e00907

**Published:** 2026-04-11

**Authors:** Simona Nedelcu

**Affiliations:** Brigham and Women's Hospital, Harvard Medical School, Boston, MA, USA

**Keywords:** ischemic stroke, diabetes, antiplatelets

Dual antiplatelet therapy (DAPT) with clopidogrel and aspirin has become a central strategy in secondary stroke prevention following a series of landmark trials demonstrating benefit in patients with minor ischemic stroke and high-risk transient ischemic attack (TIA). The CHANCE trial established that 21 days of DAPT reduced recurrent stroke by 32% compared with aspirin alone in Chinese patients presenting within 24 h of symptom onset [1]. The POINT trial similarly demonstrated a 25% reduction in ischemic events at 90 days, though with increased major bleeding [2]. A pooled analysis of these trials confirmed that the benefit of DAPT is concentrated within the first 21 days after the index event [3], suggesting that early initiation is a critical determinant of benefit.

Clopidogrel requires hepatic conversion into its active form, but this pathway is known to be inefficient among those with a CYP2C19 loss-of-function gene variant. This raises the possibility that some patients may not receive the benefit of DAPT if they harbor this gene variant. Ticagrelor in contrast to clopidogrel is not dependent on CYP2C19 for activation, making its clinical efficiency independent of CYP2C19 genotype. Approximately 30–35% of Europeans and up to 60% of East Asians carry CYP2C19 loss of function alleles, which impair clopidogrel's conversion to its active metabolite but do not affect ticagrelor's efficacy. An alternative DAPT combination was studied in the THALES trial, which evaluated ticagrelor plus aspirin versus aspirin alone in patients with acute mild to-moderate ischemic stroke (NIHSS ≤5) or high-risk TIA. This study demonstrated that ticagrelor-aspirin reduced the composite of stroke or death at 30 days, though at the cost of increased severe bleeding (0.5% vs 0.1%). Notably, an exploratory analysis confirmed consistent benefit in patients with moderate stroke (NIHSS 4–5), with no further increase in intracranial hemorrhage compared to those with milder deficits [4]. CHANCE-2 then showed that ticagrelor-aspirin was superior to clopidogrel-aspirin in CYP2C19 loss-of-function carriers, underscoring that the effectiveness of DAPT is not uniform across all patients, but may depend on underlying biology [5].

ATAMIS trial meaningfully extended this knowledge by moving beyond the classic minor-stroke population, and showing that DAPT with clopidogrel plus aspirin reduces early neurological deterioration (END) at 7 days in patients with mild-to-moderate ischemic stroke (NIHSS 4–10) presenting within 48 h, a population previously underrepresented in antiplatelet trials [6]. In the March 2026 issue of Neurotherapeutics, Hou and colleagues take the next logical step, asking whether diabetes mellitus (DM), a common and mechanistically relevant comorbidity, modifies the response to DAPT. Their post hoc analysis suggests that patients with DM derived statistically significant benefit from DAPT in reducing END (5.2% vs 8.8%; adjusted risk difference, −4.1%; P = 0.04), whereas those without DM did not reach statistical significance (4.6% vs 6.1%; adjusted risk difference, −1.9%; P = 0.07). Importantly, no significant interaction between DM status and treatment effect was observed (P = 0.38), and safety outcomes were comparable across subgroups [7].

This study by Hou and colleagues [7] raises the important hypothesis that diabetes may mark a vascular and thrombo-inflammatory phenotype in which the early hazard of stroke progression is particularly amenable to intensified antiplatelet therapy.

## Mechanistic consideration of DAPT in patients with diabetes

The biological plausibility of enhanced DAPT efficacy in diabetic patients rests on multiple converging mechanisms.

Diabetic platelets exhibit a prothrombotic phenotype characterized by calcium dysregulation through SEC61B-mediated endoplasmic reticulum leak channels, leading to elevated basal cytosolic calcium that primes platelets for activation [8].

The P2Y12 receptor, the target of clopidogrel and ticagrelor, are approximately 4-fold higher in patients with type 2 diabetes compared with healthy subjects. P2Y12 receptors in diabetic platelets exhibit constitutive activation even in the absence of ADP stimulation. This phenomenon is driven by a high glucose-reactive oxygen species-nuclear factor-κB pathway that increases receptor expression in megakaryocytes [9,10]. The constitutively active state of P2Y12 may explain why diabetic patients require more potent P2Y12 inhibition, and why dual blockade with both aspirin and a P2Y12 antagonist may be particularly effective.

Beyond platelet hyperreactivity, diabetes affects clopidogrel pharmacokinetics. Exposure to clopidogrel's active metabolite is approximately 40% lower in diabetic patients due to altered hepatic metabolism and increased carboxylesterase 1 activity that diverts clopidogrel toward inactive metabolites [11].

These pharmacokinetic considerations have important implications for the study presented by Hou and colleagues [7]. The enhanced benefit of DAPT in diabetic patients may reflect the need for dual pathway inhibition to overcome both reduced clopidogrel efficacy and heightened baseline platelet reactivity. The synergistic combination of aspirin (blocking thromboxane A2 synthesis) and clopidogrel (inhibiting ADP-mediated P2Y12 activation) may be necessary to achieve adequate platelet inhibition in diabetic patients.

## Diabetes and response to DAPT

The question of whether diabetes modifies DAPT efficacy has been examined across antiplatelet strategies. In the cardiovascular literature, the THEMIS-PCI trial demonstrated that ticagrelor plus aspirin yielded favorable net clinical benefit in diabetic patients with stable coronary artery disease and prior percutaneous coronary intervention, with consistent effects across diabetes duration and HbA1c levels [12]. The PLATO sub-study similarly showed that ticagrelor reduced ischemic events compared with clopidogrel in acute coronary syndrome patients irrespective of diabetic status [13]. However, recent data from the INSPIRES trial present a more nuanced picture: DAPT reduced recurrent stroke in patients without diabetes and those with newly diagnosed diabetes, but not in those with established diabetes history [14]. This finding highlights the heterogeneity within diabetic populations and the potential influence of disease duration and glycemic control on treatment response.

Given the pharmacokinetic limitations of clopidogrel in diabetes, ticagrelor, which does not require hepatic conversion, may theoretically offer advantages in diabetic patients, though this hypothesis requires prospective validation. Current guidelines recommend DAPT with either clopidogrel-aspirin or ticagrelor-aspirin for minor-to-moderate ischemic stroke, with ticagrelor-aspirin preferred in CYP2C19 loss-of-function carriers.

## Future directions

The clinical implications of this analysis by Hou and colleagues [7] extend beyond the specific findings. First, it reinforces that DAPT remains a reasonable therapeutic strategy across the spectrum of acute ischemic stroke patients, including those with moderate deficits and comorbid diabetes. Second, it underscores the importance of identifying patient subgroups who may derive enhanced benefit from intensive antiplatelet therapy. Third, the reassuring safety profile in diabetic patients, who are at baseline increased risk for both thrombotic and hemorrhagic complications, supports the feasibility of DAPT in this population.

Future research should prospectively evaluate whether diabetes status, or more refined metabolic phenotypes, can guide antiplatelet therapy selection in acute ischemic stroke. Head-to-head comparisons of clopidogrel-aspirin versus ticagrelor-aspirin in diabetic stroke patients would be particularly informative, given the differential mechanisms of platelet inhibition and the known impact of diabetes on clopidogrel metabolism. Novel therapeutic targets emerging from mechanistic studies, may eventually offer diabetes-specific approaches to platelet inhibition.

Until such evidence emerges, the current analysis by Hou and colleagues [7] provides valuable preliminary data suggesting that diabetic patients with acute mild-to-moderate ischemic stroke may represent a population particularly suited for DAPT, while reinforcing that this therapy remains beneficial across the broader stroke population.

## Author contribution

Simona Nedelcu. SN: Conceptualization, Writing. SN: Corresponding Author.

## Declaration of competing interest

The authors declare the following financial interests/personal relationships which may be considered as potential competing interests: Simona Nedelcu reports was provided by Brigham and Women's Hospital. Simona Nedelcu reports a relationship with Brigham and Women's Hospital that includes: If there are other authors, they declare that they have no known competing financial interests or personal relationships that could have appeared to influence the work reported in this paper.
